# Deficyt Kortyzolu, Jako Rzadka Przyczyna Cholestazy Niemowlęcej

**DOI:** 10.34763/devperiodmed.20182203.280283

**Published:** 2018-10-04

**Authors:** Patryk Lipiński, Karolina Kot, Irena Jankowska, Mieczysław Szalecki

**Affiliations:** 1 Klinika Gastroenterologii, Hepatologii, Zaburzeń Odżywiania i Pediatrii, Instytut ,,Pomnik – Centrum Zdrowia Dziecka’’, Warszawa, Polska; 2 Klinika Endokrynologii i Diabetologii, Instytut ,,Pomnik – Centrum Zdrowia Dziecka’’, Warszawa, Polska; 3 Wydział Lekarski i Nauk o Zdrowiu, UJK, Kielce, Polska

**Keywords:** kortyzol, cholestaza, wielohormonalna niedoczynność przysadki, zespół przerwanej ciągłości szypuły przysadki, rodzinny niedobór glikokortykosteroidów, cortisol, cholestasis, multiple pituitary hormone deficiency, pituitary stalk interruption syndrome, familial glucocorticoid deficiency

## Abstract

Deficyt kortyzolu stanowi rzadką przyczynę cholestazy niemowlęcej.

Celem pracy było przedstawienie patogenezy cholestazy w przebiegu deficytu kortyzolu oraz

charakterystyka wybranych zaburzeń towarzyszących deficytowi.

## Abstract (en)

Cortisol deficiency constitutes a rare cause of neonatal cholestasis.

The aim of this manuscript was to present the pathogenesis of cortisol deficiency in neonatal cholestasis.

The authors also present the characteristics of selected disorders resulting in cortisol deficiency.

## Wstęp

Zgodnie z najnowszymi (2017 rok) rekomendacjami Europejskiego i Północnoamerykańskiego Towarzystwa Gastroenterologii, Hepatologii i Żywienia Dzieci (ESPGHAN i NASPGHAN), cholestazę rozpoznaje się, gdy stężenie bilirubiny bezpośredniej w surowicy przekracza wartość 1 mg/dl, niezależnie od stężenia bilirubiny całkowitej [1]. Jednakże, u dzieci mogą występować przypadki cholestazy przebiegającej bez podwyższenia stężenia bilirubiny bezpośredniej (postać bezżółtaczkowa). Istotne znaczenie dla rozpoznania cholestazy ma wówczas stwierdzenie podwyższonego stężenia kwasów żółciowych w surowicy i/lub aktywności gamma-glutamylotranspeptydazy (γ-GT).

Diagnostyka różnicowa cholestazy jest trudna, z uwagi na mnogość przyczyn. Rokowanie uzależnione jest od postawionego rozpoznania i dotyczy nie tylko postępu uszkodzenia wątroby, ale również objęcia procesem chorobowym innych narządów i układów, w tym ośrodkowego układu nerwowego. Prawidłowo postawione rozpoznanie pozwala na szybkie wdrożenie właściwego leczenia.

## Kortyzol a cholestaza

Pierwszy opis przypadku cholestazy związanej z deficytem kortyzolu pojawił się już w 1956 roku i dotyczył niemowlęcia z wrodzoną wielohormonalną niedoczynnością przysadki (WNP). Dotychczas, w literaturze opisano ok. kilkadziesiąt przypadków niemowląt z cholestazą i WNP [2, 3, 4, 5, 6, 7, 8, 9].

Patomechanizm cholestazy w przebiegu WNP jest złożony, jednak najprawdopodobniej główną rolę odgrywa deficyt kortyzolu na skutek niedostatecznego wydzielania przysadkowej adrenokortykotropiny (ACTH), czyli wtórna niedoczynność kory nadnerczy [9].

Kortyzol jest hormonem biorącym udział w regulacji syntezy i transportu kwasów żółciowych, m.in. poprzez udział w regulacji transkrypcji genów kodujących białka BSEP, MDR3 oraz MRP2, pełniących funkcje transporterów kwasów żółciowych (ryc. 1) [10]. Grammatikopoulos i wsp. potwierdzili powyższe tezy, badając bioptaty uzyskane podczas biopsji wątroby u pacjentów z cholestazą w przebiegu niedoczynności przysadki, uzyskując zmniejszoną ekspresję białek BSEP, MDR3 oraz MRP2 [11]. Co więcej, w badaniach na szczurach, wykazano że adrenalektomia (i w jej efekcie deficyt kortyzolu) prowadzą do zmniejszenia przepływu żółci; z kolei suplementacja glikokortykosteroidów przywraca prawidłowy przepływ żółci [12, 13].

Kortyzol oddziałując na niedojrzałe hepatocyty stymuluje ich różnicowanie w komórki wewnątrzwątrobowych dróg żółciowych. Ponadto, pobudzając kanały HCO3^-^/Cl^-^ za pośrednictwem receptora glikokortykoidowego, wywiera dodatkowy efekt żółciopędny [2].

**Ryc. 1:**
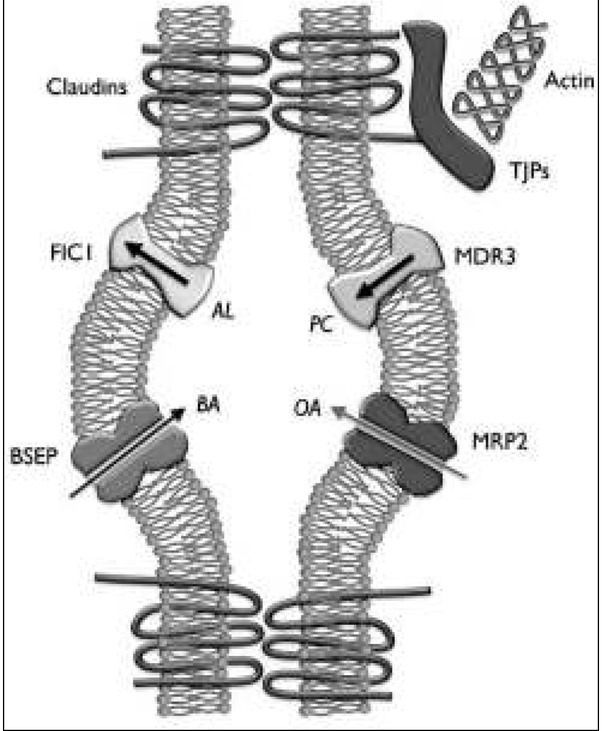
Schemat transportu kwasów żółciowych [25, opublikowano za zgodą autora]. Objaśnienia: AL – aminofosfolipidy, PC – fosfatydylocholina, BA – kwasy żółciowe, OA – aniony organiczne, FIC1 – pompa odpowiadająca za przezbłonowy transport amino-fosfolipidów, BSEP – pompa eksportująca sole kwasów żółciowych, MDR3 – przenośnik fosfatydylocholiny, MRP2 – pompa transportująca kwasy żółciowe do krążenia ogólnego. Fig. 1. Schematic presentation of bile acid transport [25]. AL – aminophospholipids, PC – phosphatidylocholine, BA – bile acids, OA – organic anions, FIC1 – aminophospholipid transporter, BSEP – bile salt export pump, MDR3 – multidrug resistance protein 3, MRP2 – multidrug resistance associated protein.

W patogenezie cholestazy w przebiegu WNP bierze się również pod uwagę znaczenie niedoboru hormonu wzrostu (GH) oraz hormonu tyreotropowego (TSH) [2, 3, 4, 5, 6, 7, 8, 9]. Zarówno GH, jak i TSH również uczestniczą w regulacji transkrypcji genów syntetyzujących biał-ko MRP2 [14]. Istnieją doniesienia o potwierdzonym w badaniu histologicznym ustąpieniu cech uszkodzenia hepatocytów i wewnątrzwątrobowych dróg żółciowych po wprowadzeniu terapii substytucyjnej hormonem wzrostu i preparatem L-tyroksyny [2]. Jednakże, jak dotąd nie opisano w literaturze przypadku niemowlęcia z cholestazą w przebiegu izolowanej somatotropinowej niedoczynności przysadki. Z kolei, przedłużająca się żółtaczka po urodzeniu, może być jednym z objawów sugerujących niedoczynność tarczycy, jednakże hiperbilirubinemia w tym przypadku przebiega z przewagą bilirubiny pośredniej [15].

Niedobór kortyzolu w okresie niemowlęcym zdarza się rzadko, a przyczyny deficytu kortyzolu mogą być wrodzone lub nabyte i mogą prowadzić do uszkodzenia nadnerczy – pierwotna niedoczynność nadnerczy, lub dotyczyć przysadki i podwzgórza – wtórna i trzeciorzę-dowa niedoczynność nadnerczy.

Niedobór kortyzolu stwierdzamy między innymi we wrodzonej wielohormonalnej niedoczynności przysadki, izolowanym niedoborze ACTH, urazie przysadki, po glikokortykoterapii, we wrodzonym przeroście kory nadnerczy, wrodzonej hipoplazji nadnerczy (zespół DAX-1), adrenoleukodystrofii (X-ALD), wylewach do nadnerczy, kiedy zniszczeniu ulega ponad 90% miąższu nadnerczy oraz w rodzinnym niedoborze glikokortykoidów spowodowanym niewrażliwością nadnerczy na ACTH.

## Wielohormonalna niedoczynność przysadki

W etiologii wielohormonalnej niedoczynności przysadki rozpatruje się wpływ czynników środowiskowych i genetycznych. W przypadku wrodzonego charakteru schorzenia, ujawnienie objawów choroby może nastąpić w okresie niemowlęcym, a nawet wczesnodziecięcym. Częstość występowania ocenia się na około 1 na 50 000 urodzeń, z czego w 10 do 35% obserwuje się współwystępowanie cholestazy [2]. Wrodzona niedoczynność przysadki dotyczy najczęściej kilku osi hormonalnych, jednakże możliwe są sporadyczne przypadki izolowanej niedoczynności przysadki w zakresie np. wydzielania ACTH, mówimy wtedy o izolowanym niedoborze ACTH [3].

Poza cholestazą, objawami które mogą nasuwać podejrzenie WNP, są epizody nawracającej hipoglikemii. W zależności od stopnia niedoboru hormonów przysadkowych (GH, TSH) oraz kortyzolu, hipoglikemia może manifestować się niepokojem, zaburzeniami oddychania czy drgawkami już w 1. dobie życia dziecka [2, 3, 4, 5, 6, 7, 8, 9].

O wielohormonalnej nieodczynności przysadki warto także pomyśleć w przypadku obecności zaburzeń rozwojowych oczu i linii pośrodkowej ciała (małoocze/bezocze, rozszczep wargi i/lub podniebienia, hipoplazja nerwów wzrokowych, hipoplazaja/agenezja ciała modzelowatego) oraz nieprawidłowości dotyczących rozwoju narządów płciowych (u chłopców mikropenis czy wnętrostwo) [2, 3, 4, 5, 6, 7, 8, 9, 16].

Podstawę rozpoznania WNP stanowią badania hormonalne. Obecność wady strukturalnej w okolicy podwzgórzowo-przysadkowej zwiększa prawdopodobień-stwo wystąpienia WNP [6]. Na uwagę zasługuje zespół przerwanej ciągłości szypuły przysadki (ang. *pituitary stalk interruption syndrome*, PSIS), w którym obrazowanie metodą rezonansu magnetycznego pozwala uwidocznić przerwaną ciągłość szypuły przysadki, której zwykle to-warzyszy brak lub ektopia tylnego płata przysadki oraz hipoplazja przedniego płata przysadki. W pracy Mauvaix i wsp., opublikowanej w 2016 roku, będącej retrospektywną analizą pacjentów z PSIS rozpoznanym przed ukończeniem 1. roku życia, żółtaczka cholestatyczna była obecna u 31% pacjentów, i obok epizodów hipoglikemii stanowiła jeden z najwcześniejszych objawów klinicznych sugerujących niedoczynność przysadki. Co więcej, stężenie kortyzolu w surowicy w grupie pacjentów z cholestazą było znacznie bardziej obniżone niż w grupie bez cholestazy [16].

W diagnostyce wykorzystuje się również badania molekularne. Genezy wad strukturalnych OUN z to-warzyszącym zespołem PSIS upatruje się mutacjach genów: *TGIF*, *SHH*, *CDON*, *GPR161*, *PROKR2*, *HESX1*, *OTX2*, *LHX3/LHX4*, *SIX6*, *PTX2*; w izolowanym niedoborze ACTH – *TBX19*. Powiększenie przysadki może występować u pacjentów z mutacją w genie *PROP1* [16, 17, 18]. Badanie histologiczne bioptatu wątroby nie jest patognomoniczne [2, 3, 4, 5, 6, 7, 8, 9]. Wczesne rozpoznanie i wdrożenie leczenia substytucyjnego powoduje całkowite wyleczenie, w tym ustąpienie cholestazy.

## Rodzinny niedobór glikokortykosteroidów

W diagnostyce różnicowej cholestazy niemowlęcej przebiegającej z deficytem kortyzolu i prawidłowym/pod-wyższonym stężeniem ACTH (pierwotna niedoczynność kory nadnerczy) oprócz zaburzeń peroksysomalnych, lizosomalnych i mitochondrialnych należy brać pod uwagę rodzinny niedobór glikokortykosteroidów (ang. *familial glucocorticoid deficiency*, FGD) [4, 7, 19, 20, 21, 22, 23, 24].

Rodzinny niedobór GKS jest heterogenną, bardzo rzadką chorobą związaną z deficytem glikokortyko-steroidów (zwłaszcza kortyzolu) oraz opornością kory nadnerczy na ACTH. Dziedziczy się autosomalnie rece-sywnie i w większości przypadków związany jest z mutacją genu kodującego receptor ACTH, u pozostałych mutacje mogą dotyczyć rejonu regulatorowego genu kodującego receptor ACTH lub innych czynników odpowiedzialnych za różnicowanie kory nadnerczy (geny *MC2R, MRAP, MCM4, NNT, AAAS* [19, 20, 21, 22, 23, 24].

Objawy choroby ujawniają się zwykle w okresie noworodkowym lub niemowlęcym pod postacią epizodów hipoglikemii, cholestazy i małego przyrostu masy ciała [4, 7, 19, 20, 21, 23, 24]. W badaniu przedmiotowym zwraca uwagę hiperpigmentacja skóry i błon śluzowych, bę-dąca efektem nadmiernego wydzielania ACTH, która może się ujawnić w toku dalszej diagnostyki. Leczenie substytucyjne powoduje ustąpienie objawów, w tym cholestazy.

## Badania hormonalne

Warto podkreślić różnice w stężeniach hormonów (kortyzolu, a szczególnie ACTH) w opisywanych wyżej patologiach. W WNP mamy do czynienia z wtórną niedoczynnością kory nadnerczy, a więc niskiemu stężeniu kortyzolu w surowicy towarzyszy niskie stężenie ACTH w osoczu. Z kolei, w FGD mamy do czynienia z pierwotną niedoczynnością kory nadnerczy, a więc niskiemu stężeniu kortyzolu w surowicy towarzyszy prawidłowe stężenie ACTH w osoczu.

Normy stężeń kortyzolu w surowicy w godzinach porannych wynoszą ok. 5-25 μg/dl. W zaburzeniach endokrynologicznych przebiegających z faktycznym defi-cytem kortyzolu zwykle obecne są objawy kliniczne hipokortyzolemii, w tym m.in. epizody hipoglikemii. Należy pamiętać, że u niemowląt dolna granica normy stężenia kortyzolu w surowicy, jest niższa [26, 27]. Największą wartość diagnostyczną u niemowlęcia z podejrzeniem endokrynopatii ma oznaczanie stężenia kortyzolu właśnie w trakcie hipoglikemii. Ze względu na niewykształcony jeszcze rytm dobowy wydzielania kortyzolu u niemowląt, oznaczanie stężenia kortyzolu i ACTH nie musi ograniczać się do godzin porannych.

## Podsumowanie

U niemowląt z cholestazą o nieustalonej etiologii należy wziąć pod uwagę deficyt kortyzolu i w związku z tym uwzględnić badanie stężenia kortyzolu (badanie powinno się wykonywać rano, na czczo) w panelu wykonywanych badań. Co więcej, takie postępowanie jest zalecane w najnowszych rekomendacjach Europejskiego i Północnoamerykańskiego Towarzystwa Gastroenterologii, Hepatologii i Żywienia Dzieci (ESPGHAN i NASPGHAN) dotyczących cholestazy niemowlęcej.
